# Prevalence and Recovery From Olfactory and Gustatory Dysfunctions in Covid-19 Infection: A Prospective Multicenter Study

**DOI:** 10.1177/1945892420930954

**Published:** 2020-06-12

**Authors:** Eléonore Chary, Florent Carsuzaa, Jean-Paul Trijolet, Anne-Laure Capitaine, Mariam Roncato-Saberan, Kevin Fouet, France Cazenave-Roblot, Mélanie Catroux, Caroline Allix-Beguec, Xavier Dufour

**Affiliations:** 1 ORL—Head and Neck Surgery, University Hospital of Poitiers, Poitiers, France; 2 ORL—Head and Neck Surgery, Saint Louis Hospital, La Rochelle, France; 3 Infectious Diseases, Saint Louis Hospital, La Rochelle, France; 4 Infectious Diseases, University Hospital of Poitiers, Poitiers, France; 5 Clinical Research Department, Saint Louis Hospital, La Rochelle, France

**Keywords:** Covid-19, Anosmia, Ageusia, Olfactory disorders, Gustatory disorders, Recovery, SARS-CoV-2

## Abstract

**Background:**

Covid-19 is defined by an association of multiple symptoms, including frequently reported olfactory and gustatory disorders.

**Objective:**

The main purpose of this study was to analyze the prevalence of these neurosensory impairments in patients with Covid-19, and to assess short-term recovery.

**Methods:**

We performed a multicenter case series study during the Covid-19 epidemic. All patients presenting a RT-PCR-confirmed SARS-CoV-2 infection were included, whether hospitalized or treated at home. To analyze the prevalence and features of olfactory and gustatory dysfunctions, a phone interview was conducted 5 days after the positive PCR result. The questionnaire was submitted again 10 days later to patients having reported olfactory and gustatory disorders, in order to assess their recovery.

**Results:**

115 patients were included in our study. 81 patients (70%) reported olfactory and gustatory disorders without nasal obstruction or rhinorrhea. These impairments were more frequently reported in the female population, young people, and house-bound patients with mild symptomatic forms. Short-term recovery assessed at Day 15 was complete for 64% of the patients, and incomplete in 33%. Median recovery time was 15 days (4–27 days) after olfactory or gustatory symptom onset.

**Conclusion:**

Olfactory and gustatory dysfunctions related to Covid-19 are frequently reported and prevalent in mild symptomatic forms of the disease. Recovery in most cases seems rapid and complete.

## Introduction

The new coronavirus disease 2019 (Covid-19) is a highly contagious zoonosis resulting from the SARS-CoV-2 infection, which is transmitted from human to human by respiratory secretions. This viral pandemic is ongoing from December 2019. It emerged from China and quickly spread to the entire world. This virus, belonging to the coronavirus family, can result in various clinical presentations of the disease, from common rhinorrhea to severe acute respiratory syndrome. According to several clinical studies, the most prevalent symptoms of Covid-19 consist of fever, cough, dyspnea, myalgia, headaches, diarrhea, sore throat and rhinorrhea.^1–3^ Similarly to other kinds of coronavirus such as severe acute respiratory syndrome (SARS) coronavirus, this virus seems to have a particular tropism for the nervous structures of the olfactory bulb.^4,5^ According to several epidemiological studies,^6–10^ it could cause olfactory and gustatory dysfunctions in many infected patients, especially those with mild to moderate forms of the disease. Neuro-sensory impairment could be a warning sign or an inaugural symptom of Covid-19.^7,8,10^ Tracking and isolation of mildly symptomatic patients is essential to stemming the viral spread, of which the contagiousness is exponential. Description of precursor or inaugural symptoms appears necessary to improve the screening and diagnosis of mild symptomatic forms. In addition, detection of these olfactory and gustatory dysfunctions could be helpful to forecast the disease’s course. The progression of olfactive loss in Covid-19 remains unknown. In usual post-viral anosmia, 30 to 50% of the patients report complete recovery of their olfaction, while 50% show permanent anosmia.^11,12^ Anosmia is a disabling disorder that can severely affect quality of life. Indeed, depression syndromes, domestic incidents and eating disorders related to these sensorial dysfunctions have been reported.^11–13^ Olfactory reeducation may yield significant improvement of olfactory abilities in these kinds of post-viral anosmia, stimulating the neuronal regeneration of olfactory bulb cells.^14^ It consequently appears essential to program early screening of the Covid-19-related olfactory disorders, in order to prescribe the earliest possible reeducation. Screening of rhinologic otolaryngological impairments appears essential to diagnosis of Covid-19, especially in mildly symptomatic patients. For this purpose, we decided to assess the prevalence of olfactory and gustatory disorders in infected patients and to analyze their olfaction recovery.

## Methods

A prospective multicenter case series study was conducted between March 25^th^ and April 18th 2020. The study protocol was declared to the National Commission for Data Protection (CNIL), reference MR-004. It was a prospective study based on data, not directly involving clinical care, and therefore did not require personal protection committee approval (INDS number MR 1116270320). Patients received clear and loyal oral information. Their oral consent was obtained during phone interviews, and a written explanation could be sent to them by e-mail.

### Subjects and Settings

All the patients with laboratory-confirmed Covid-19 infection (using reverse transcription polymerase chain reaction, RT-PCR) were included, whether subsequently hospitalized or house-bound. RT-PCR was performed on nasopharyngeal samples using SEEGENE® reagent (Allplex 2019 nCov Assay- Eurobio Scientific) with LightCycler®. Exclusion criteria were as follows: underage patient <18 years, patients unable to fill out the questionnaire (patients with dementia, confused patients, patients non-reachable by phone, patients in the intensive-care unit at the time of the study), non-native French speakers or deaf patients who did not use a hearing aid, and patients with olfactory or gustatory dysfunctions known before the epidemic.

The main purpose of this study was to investigate the prevalence and to characterize the occurrence of subjective olfactory and gustatory disorders in patients with laboratory-confirmed Covid-19 infection. Patients matching the inclusion criteria were interviewed by phone at Day 5 following the positive RT-PCR result.

### Clinical Outcomes

Clinical data extracted from interrogation carried out during initial care was collected on an electronic Case Report Form (CRF). This CRF included age, gender, medical and surgical history, otolaryngological comorbidities, onset of general symptoms and main symptoms presented during the first two days of the disease, among the following ones: cough, fever, myalgia, headaches, diarrhea, rhinorrhea. We also looked in the patients’ medical records for the main reason for hospitalization and possible resuscitation transfer. Lastly, the phone interview entailed a search for qualitative or quantitative olfactory and/or gustatory disorders, not related to nasal obstruction or rhinorrhea.

### Olfactory and Gustatory Outcomes

**Figure 1.**
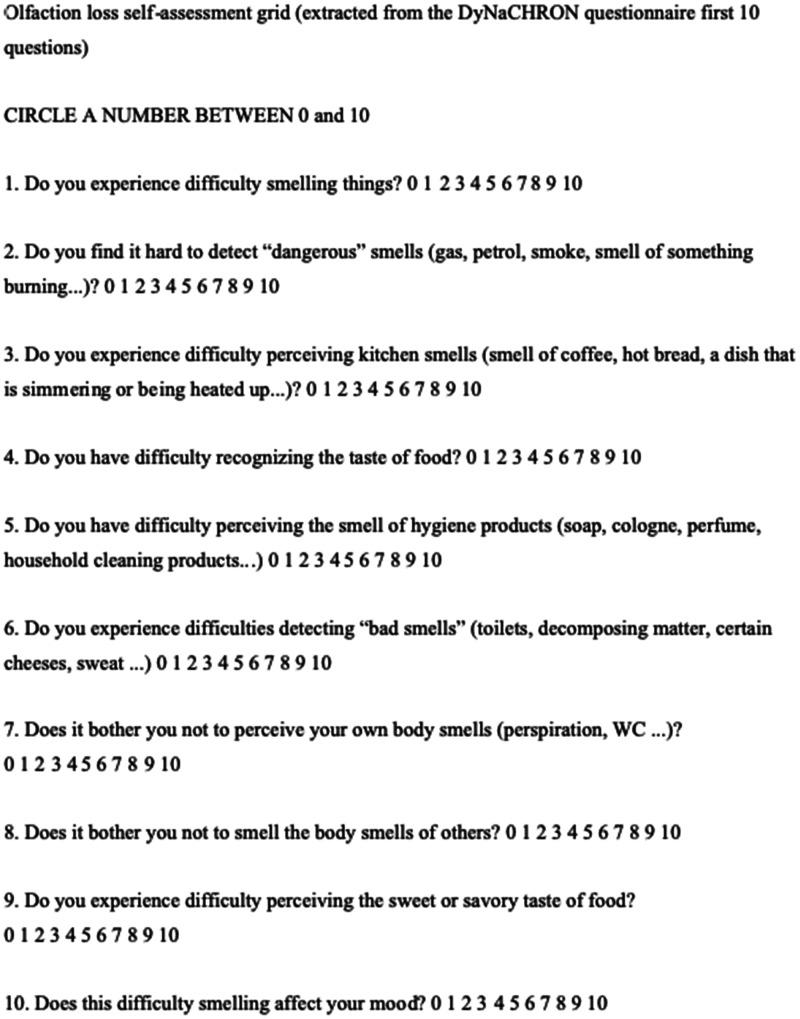
DyNaCHRON questionnaire translated.

In order to characterize and assess the severity of these sensory impairments, we used a 10-item questionnaire. These items were adapted from the DyNaCHRON questionnaire.^15^ Only those concerning smell and taste were used in our study. Each item was scored from 0 to 10. Zero was equivalent to absence of symptom and 10 to maximum symptom. A translated version is available in English (Figure 1). The secondary aim of this study was to estimate short-term recovery from these sensory disorders at Day 15 after RT-PCR. This was achieved by conducting a second phone interview, ten days later, still using the DyNaCHRON questionnaire. Complete recovery was defined by a score between 0 and 5 out of 100, at Day 15. Partial recovery at Day 15 was defined by a score between 5 and the first score obtained at Day 5, out of 100. Lack of recovery at Day 15 was defined by a score equal or superior to the first score obtained at Day 5.

### Statistical Methods

Excel® Software was used to perform the statistical analyses. Data were collected in Excel® tables, including statistical descriptions according to the following terms: For quantitative variables: median, minimal values, maximal values; for qualitative variables: absolute values and percentages (missing data were excluded from denominator). The average equality hypothesis was tested for quantitative variables. The cohort homogeneity hypothesis was checked for qualitative variables. Concerning quantitative variables, after variance equality and normal distribution of the values were checked, we used the Student t-Test. In case of variance inequality or non-Gaussian distribution of the values, a non-parametric Mann-Whitney/Wilcoxon test was performed. For the analyses of qualitative variables, a Chi-square Test was performed, or a Fisher Test in case of small-size samples. A level of p < 0.05 was used to determine statistical significance, with a bilateral alpha risk of 5%.

## Results

A total of 115 patients were included between March 25th and April 18th 2020. Patient characteristics, main symptoms presented in the first two days of the disease and rate of hospitalizion (with or without resuscitation transfer) are described in Table 1. 81 patients (70%) reported qualitative or quantitative olfactory or gustatory disorders, without nasal obstruction or rhinorrhea. There was no statistically significant association between general symptoms of Covid-19 (fever, cough, sore throat, myalgia, headaches, diarrhea, rhinorrhea) and olfactory or gustatory dysfunctions. The occurrence of olfactory and gustatory disorders, without any nasal obstruction or rhinorrhea, was significantly more prevalent in young patients (median 42 y.o vs 55 y.o in patients with no impairments) and in the female population (77% vs 56% in patients with no sensory impairments, *p = 0.027*). The rate of severe forms of Covid-19 requiring intensive care was higher in patients with no neuro-sensory impairments (*p = 0.043*) (Table 2). Median onset of the olfactory and gustatory impairments was 2 days after onset of the first symptoms (ranged from −3 (3 days before) to +7 (7 days after)). The main symptoms were the association of anosmia and hypogeusia (33%), association of anosmia and ageusia (32%) and isolated anosmia (15%). Other forms of sensory dysfunctions (hyposmia and hypogeusia: 6%; isolated hyposmia: 5%; isolated ageusia: 2%; isolated hypogeusia: 5%; association of hyposmia and ageusia: 1%) were less represented. Olfactory and gustatory impairments were associated in 72% of cases. If most olfactory and gustatory disorders was represented by quantitative impairments, qualitative dysfunctions were also found in a small proportion. Thus, 4 cases of parageusia were reported, as well as 3 cases of cacogeusia, 4 cases of phantosmia and 5 cases of cacosmia. The median RT-PCR cycle following symptom onset was 5 days (0 to 17), with no significant difference between patients with neuro-sensitive disorders and the others.

**Table 1.** Characteristics of Population.

Population	n = 115
Median age [min–max]	47 [20–83]
Women	81 (70%)
Cough	49 (43%)
Fever	70 (61%)
Odynophagia	21 (18%)
Myalgia	57 (50%)
Headache	62 (54%)
Diarrhea	20 (17%)
Rhinitis	25 (22%)
Hospitalization	27 (24%)
Intensive Care Unit	4 (4%)

**Table 2.** Clinical Characteristics Depending on Presence or Absence of Olfactory and Gustatory Dysfunctions.

	No Troubles (n = 34)	Olfactory and Gustatory Dysfunctions (n = 81)	p value
Median age [min–max]	55 [27–79]	42 [20–83]	0,003*
Women	19 (56%)	62 (77%)	0,027*
Cough	15 (44%)	34 (42%)	0,873
Fever	25 (74%)	45 (56%)	0,083
Odynophagia	7 (21%)	14 (17%)	0,577
Myalgia	17 (50%)	40 (49%)	1
Headache	17 (50%)	45 (56%)	0,54
Diarrhea	6 (18%)	14 (17%)	0,985
Rhinitis	8 (24%)	17 (21%)	0,788
Hospitalization	13 (38%)	14 (17%)	0,016*
Intensive care unit	3 (9%)	1 (1%)	0,043*
Median score Dynachron (/100)	0	80 [10-97]	0,001*

*denotes significant values.

**Figure 2.**
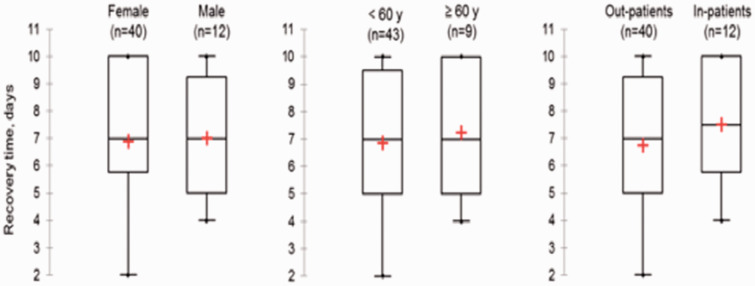
Analysis of the median time of full recovery on D5 or D15 from RT – qPCR time according to patient characteristics.

**Figure 3.**
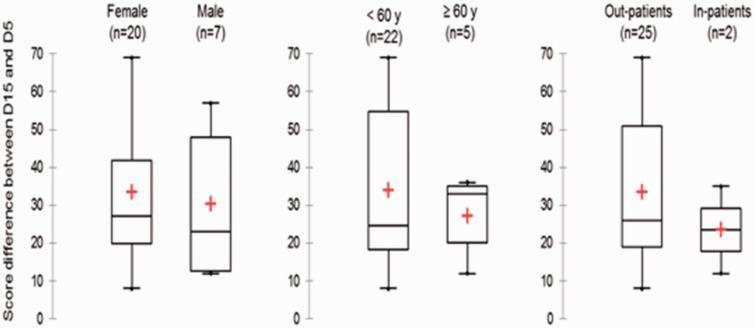
Analysis of the medians of the differences between questionnaire scores on D5 or D15 from the RT – qPCR time according to patient characteristics.

**Figure 4.**
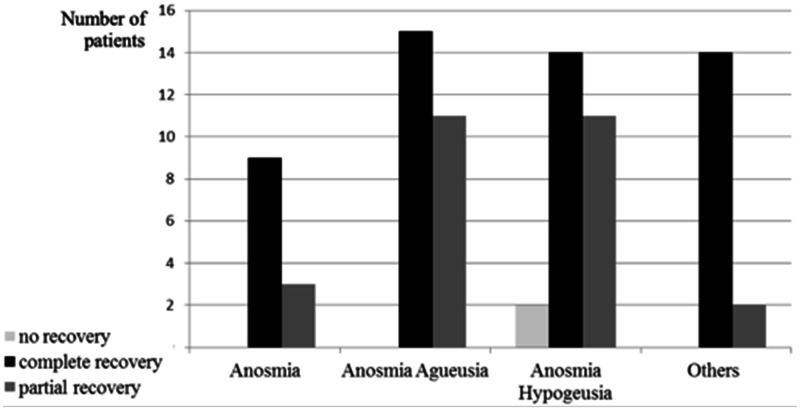
Description of recovery according to neurosensory characteristics.

Concerning recovery, complete recovery was observed in 64% of the cases. Partial recovery was noted in 33%, and lack of recovery in 2 patients (3%), at the Day 15 assessment. The median recovery period between the two assessments was 7 days. Therefore, the median recovery’s period following olfactory and gustatory symptoms onset was 15 days (4 to 27). There was no significant difference in the cohort regarding age, gender or care (Table 3). There was no significant difference in the complete recovery duration related to age, gender, need for hospitalization (Figure 2). About partial recovery, the median of points lost between the two assessments, corresponding to improved quality of life, was 26 points (ranging from 8 to 69). There was no significant difference related to age, gender or need for hospitalization (Figure 3). Neuro-sensory categorization’s data and data concerning complete, partial or lack of recovery are described in Figure 4. They were not analyzed because of the small sample size.

**Table 3.** Characteristics of Patients With Partial or Complete Recovery.

	Complete Recovery (n = 52)	Partial Recovery (n = 27)	p
Median Age [min–max]	41,5 [20–80]	49 [20–83]	0,74
Women	40 (77%)	20 (74%)	0,779
Hospitalization	12 (23%)	2 (7%)	0,084

## Discussion

Since the beginning of Covid-19 pandemic, studies have reported olfactory and taste disorders related to this infection. The high rate of these symptoms (70%) in our population is comparable to outcomes from other international reports.^7–9^ This association has been found primarily demonstrated in young patients and the female population. The higher susceptibility of females to olfactory and gustatory dysfunctions may be related to the gender-related differences in the inflammatory reaction process.^16^ Another hypothesis is interference with some bias. First, the population most exposed to the virus is represented by healthcare workers (nursing staff), who are active and young people, most of them without medical history. However, nurses and physicians are often women, and that fact could partially explain our results about higher prevalence in female patients. Moreover, when this study was carried out, screening was not being performed in the general population in France. Screened patients were nursing staff with symptoms, symptomatic pregnant women in the third trimester, and suspected patients with hospitalization criteria. The high rate of olfactory and gustatory dysfunctions (70%) in our population is nonetheless comparable to outcomes from other European studies.^7–10^ On the other hand, these impairments have been less frequently reported in Asian studies. A statistically significant association between the olfactory and gustatory symptoms and the mildly symptomatic forms of the disease, which are mostly treated at home, was shown in the study by Yan et al.^17^ Severe forms of Covid-19 requiring hospitalization were not associated with olfactory or gustatory disorders. These impairments could be a major positive prognostic factor for Covid-19. However, this hypothesis could be limited in extension insofar as patients in critical care were not interviewed, due to their health status. In our study, the median onset of the olfactory and gustatory impairments was 2 days after occurrence of general symptoms (fever, cough, diarrhea, rhinorrhea, myalgia). The sensory symptoms appeared early in the course of the disease and seemed to be relatively specific. Recognition and tracking of these symptoms, at times isolated or present in mildly symptomatic patients, could help to isolate them earlier and thereby limit spread of the disease. To assess sensory recovery, we chose a questionnaire usable by phone and reproducible between patients and during the time of the study. DyNaCHRON is a validated questionnaire for global assessment of rhinologic functions.^15^ We used a short version of DyNaCHRON including only the first 10 items concerning smell and taste abilities. Indeed, the rest of the items did not seem suitable in an emergency situation. The relevance of these results might have been enhanced by using subjective olfaction tests^18^ such as the Sniffin’ Sticks tests (Burghardt®, Wedel, Germany). However, because of the patient’s contagiousness and in order to respect the protective measures implemented in our structures, we were unable to use these tests. The pathophysiological mechanisms leading to olfactory and gustatory dysfunctions in Covid-19 remain unclear. Central nervous system impairment through olfactive nerve infection has been proposed as a hypothesis explaining anosmia in SARS infection, which has many common features with SARS-CoV-2.^5^ Besides, this hypothesis was confirmed by Netland et al in rats exposed to SARS-CoV-2.^19^ After being exposed by inhalation to SARS-CoV-2, the virus was detected 60 hours later in the olfactory bulb of the rodents, and four days later it had spread to the piriform cortex. It is now suspected that the virus links to angiotensin-converting enzyme 2 (ACE2) receptor through surface proteins. This receptor is particularly expressed in respiratory epithelioma. ACE2 could be implicated in the difference of neuro-sensory symptom prevalence between Asian and European populations. According to Li et al., ACE2 variability^20^ could reduce the association between human ACE2 et the S-protein on the SRAS-CoV-2 surface. This genetic variability has been highlighted in a Chinese study.^21^ Comparison between 15 variances of quantitative expression from ACE2 gene loci suggest high polymorphism of ACE2 and its expression level in Asian and European populations. One of the original features of our study was assessment of short-term recovery of smell and taste. We observed complete recovery at Day 15 from the RT-PCR results in 64% of the cases. Median complete recovery time was 15 days (4 to 27) after the onset of the olfactory and gustatory symptoms. However, even in the partial recovery cohort, quality of life, assessed with DyNaCHRON questionnaire, was improved, with a median of lost points at Day 15 of 26 points out of 100. The duration of recovery in patients with complete recovery was not significantly related to age, gender or hospital care. Moreover, median of lost points in the partial recovery cohort was not significantly impacted by age, gender or house-bound care. A study published by Lechien et al.^7^ described a complete recovery rate of 44% within the 5 to 8 days following resolution of the general symptoms. Lastly, 72,6% of these patients recovered completely their olfactory and gustatory functions within the first 8 days following resolution of the disease. These results predominantly showing a short recovery time are consistent with the study by Kaye et al.^22^ However, because of the short study period and the limited sample size, it will be necessary to confirm these outcomes with other studies. That much said, the hypothesis suggesting short-term recovery from neurosensory impairments in Covid-19 infection appears more than plausible.

## Conclusion

Olfactory and gustatory dysfunctions related to Covid-19 have frequently been reported in Europe (70%) and should help to screen, identify and thereby quickly isolate mildly symptomatic patients from the general population. This information should be disseminated to the public in order to facilitate screening of mildly symptomatic patients. The presence of these dysfunctions could be a prognostic factor in the disease’s course. Neuro-sensory recovery seems to be fast and complete in most patients. In our study, we did not identify any predisposing factor to a slower recovery. A study monitoring patients and assessing their recovery prognostic in the medium term, especially using olfactory bulb MRI, could be interesting and useful.
